# Supplementary material for: Exploring the feasibility of using real-world data from a large clinical data research network to simulate clinical trials of Alzheimer’s disease
Source: NPJ Digit Med. 2021 May 14;4:84. doi: 10.1038/s41746-021-00452-1 (PMC8121837; doi:10.1038/s41746-021-00452-1)
Supplement: Supplementary file 1 — Supplementary Information [file 41746_2021_452_MOESM1_ESM.pdf]

Supplementary Table 1. Baseline characteristics of propensity matching variables before and after propensity score matching (PSM)

	Before PSM		After PSM	
Variable	23mg arm	10mg arm	23mg arm	10mg arm
Age (Mean)	79.05	78.53	79.05	74.07
CCI (Mean)	1.32	1.64	1.32	1.00
Gender (Female)	73.7	70.1	73.7	84.2
Race (%)				
Non-Hispanic White	65.8	35.8	65.8	42.1
Hispanic	10.5	24.6	10.5	33.2
Other	23.7	40.6	23.7	23.7

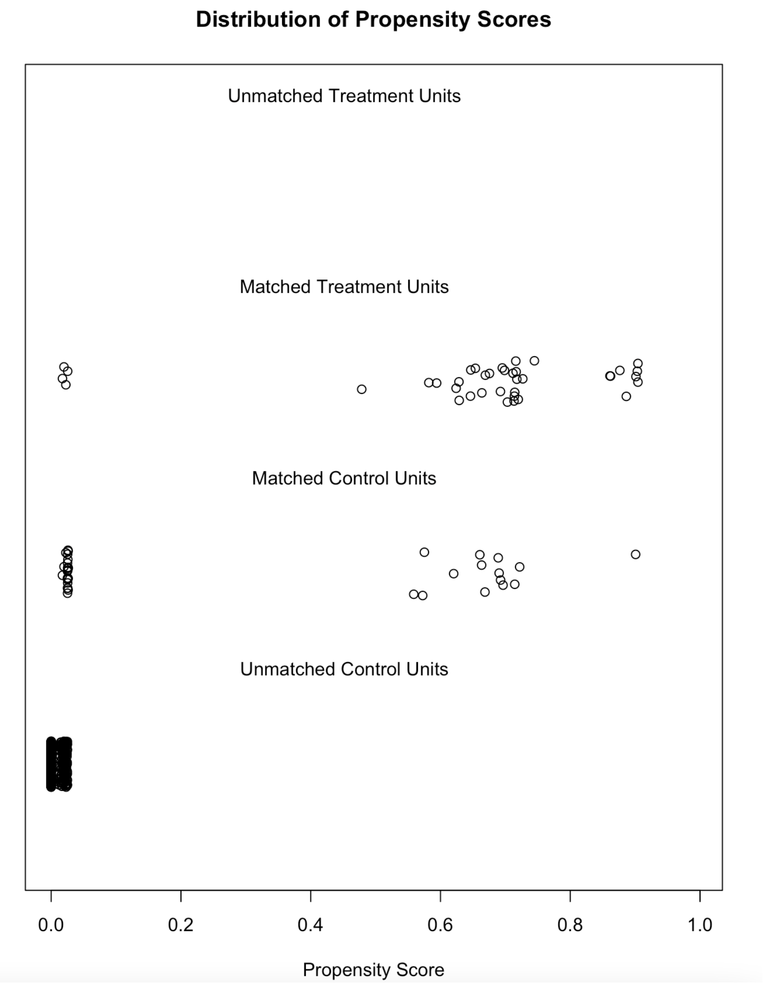

Supplementary Figure 1. Distribution of propensity scores in matched and unmatched groups from propensity score matching

Supplementary Table 2. Eligibility criteria of the original trial NCT00478205

Category	Criteria	Decomposed study traits	Computable
Inclusion	Written informed consent.	Written informed consent	N
	Patient Age range: Adult patients, 45 to 90 years of age, inclusive.	Patient Age range: Adult patients, 45 to 90 years of age, inclusive.	Y
	The caregiver must separately meet the specified inclusion/exclusion criteria for caregivers.	The caregiver must separately meet the specified inclusion/exclusion criteria for caregivers.	N
	Women must be of non-child-bearing potential (>1 year post-menopausal or surgically sterile).	Women must be of non-child-bearing potential	P
		post-menopausal	N
		surgically sterile	Y
	There must be diagnostic evidence of probable Alzheimer's disease (AD).	There must be diagnostic evidence of probable Alzheimer's disease (AD).	Y
	The patient must have been receiving Aricept at a dose of dose of 10 mg IR (or 10 mg dose of generic donepezil bioequivalent to Aricept), for at least 3 months prior to the Screening visit.	The patient must have been receiving Aricept at a dose of dose of 10 mg IR	Y
	A cranial image is required, with no evidence of focal brain disease that would account for dementia.	A cranial image is required, with no evidence of focal brain disease that would account for dementia.	N
	The patient must meet certain psychometric test criteria related to the degree of impairment of cognitive functioning	The patient must meet certain psychometric test criteria related to the degree of impairment of cognitive functioning.	N
	Health: physically healthy and ambulatory or ambulatory-aided (i.e., walker or cane); corrected vision and hearing sufficient for compliance with testing procedures, and able to read prior to disease onset.	physically healthy	N
		corrected vision and hearing sufficient for compliance with testing procedures, and able to read prior to disease onset.	N
	Clinical laboratory values must be within normal limits or, if abnormal, must be judged not clinically significant by the investigator.	Clinical laboratory values must be within normal limits	P
		Clinical laboratory, if abnormal, must be judged not clinically significant by the investigator.	P
	Specified doses of selective serotonin reuptake inhibitors (SSRIs) are allowed in the study if dosage is within approved dose range and stable for 3 months prior to Screening.	Specified doses of selective serotonin reuptake inhibitors (SSRIs) are allowed in the study if dosage is within approved dose range and stable for 3 months prior to Screening.	N
	Other medical conditions, such as hypertension and cardiac disease must be well-controlled and the patient maintained on stable doses of medications for 3 months.	well-controlled hypertension	P
		well-controlled cardiac disease	P
		patient maintained on stable doses of medications for 3 months.	P
	Patients with diabetes mellitus or risk factors for diabetes mellitus may be enrolled in the study provided that the	Patients with diabetes mellitus or risk factors for diabetes mellitus may be enrolled in the study provided that the	P

	patient's disease is stable and that there have been no recent hospitalizations for diabetes complications.	patient's disease is stable and that there have been no recent hospitalizations for diabetes complications.	
		no recent hospitalizations for diabetes complications.	Y
	Patients whose serum B12 levels at Screening are below the normal range may nonetheless be admitted to the study if they subsequently show normal levels prior to Baseline.	Patients whose serum B12 levels at Screening are below the normal range may nonetheless be admitted to the study if they subsequently show normal levels prior to Baseline.	Y
	Patients with hypothyroidism who are on a stable dose of medication for at least 12 weeks prior to Screening, have normal TSH and free T4 at Screening, and are considered euthyroid will be eligible.	Patients with hypothyroidism who are on a stable dose of medication for at least 12 weeks prior to Screening	Y
		who are on a stable dose of medication for at least 12 weeks prior to Screening	N
		have normal TSH	Y
		free T4 at Screening	Y
		are considered euthyroid will be eligible.	Y
	Concomitant Medications: Under specified circumstances, the following medications may be allowed: chronic daily benzodiazepine use, bronchodilator medications for treatment of chronic obstructive pulmonary disease (COPD), and memantine. Certain other additional prescription treatments for AD, such as other cholinesterase inhibitors, must have been discontinued for at least 3 months prior to screening.	chronic daily benzodiazepine use	Y
		bronchodilator medications for treatment of chronic obstructive pulmonary disease (COPD)	Y
		memantine	Y
		Certain other additional prescription treatments for AD, such as other cholinesterase inhibitors, must have been discontinued for at least 3 months prior to screening.	Y
	The patient must have a relative/caregiver who supervises the regular taking of the drug at the correct dose and is alert for possible side effects, unless the patient's legal guardian takes on this task.	The patient must have a relative/caregiver who supervises the regular taking of the drug at the correct dose and is alert for possible side effects, unless the patient's legal guardian takes on this task.	N
Exclusion	Patients are excluded if they are taking (a) no medication for Alzheimer's disease, (b) Aricept or bioequivalent generic donepezil at doses other than 10 mg daily, or 10 mg for less than 3 months before Screening; (c) other medications for Alzheimer's disease, except that memantine, Vitamin E, fish oil, and/or gingko biloba are allowed if doses have been stable for at least 3 months prior to the Screening visit. Patients undergoing any alternative medical techniques, such as acupuncture or acupressure, specifically for the treatment of AD are not eligible	taking (a) no medication for Alzheimer's disease	Y
		(b) Aricept or bioequivalent generic donepezil at doses other than 10 mg daily, or 10 mg for less than 3 months before Screening;	Y
		(c) other medications for Alzheimer's disease, except that memantine, Vitamin E, fish oil, and/or gingko biloba are allowed if doses have been stable for at least 3 months prior to the Screening visit.	Y

		Patients undergoing any alternative medical techniques, such as acupuncture or acupressure, specifically for the treatment of AD are not eligible	Y
	No caregiver available to meet the inclusion criteria for caregivers.		N
	Patients who have no measurable blood levels of donepezil in blood samples collected at Screening.	measurable blood levels of donepezil in blood samples collected at Screening	N
	Patients with neurological disorders that affect cognition or the ability to assess cognition but are distinguishable from Alzheimer's disease. These include, but are not limited to, Parkinson's disease, multi-infarct dementia, and dementia due to cerebrovascular disease.	Parkinson's disease	Y
		multi-infarct dementia	Y
		dementia due to cerebrovascular disease	P
	Patients with psychiatric disorders affecting the ability to assess cognition such as schizophrenia, bipolar or unipolar depression. Patients with clinically significant sleep disorders will also be excluded unless these are controlled by treatment and clinically stable for > 3 months prior to screening.	Patients with psychiatric disorders affecting the ability to assess cognition	P
		schizophrenia	Y
		bipolar	Y
		unipolar depression	Y
		Patients with clinically significant sleep disorders will also be excluded unless these are controlled by treatment and clinically stable for > 3 months prior to screening.	N
	Patients with dementia complicated by other organic disease or Alzheimer's disease with delirium.	dementia complicated by other organic disease	P
		Alzheimer's disease with delirium	Y
	Patients with drug or alcohol abuse or dependence within the past 5 years according to DSM IV criteria.	drug abuse or dependence within the past 5 years	Y
		alcohol abuse or dependence within the past 5 years	Y
	Patients with any conditions affecting absorption, distribution, or metabolism of the study medication (e.g., inflammatory bowel disease, gastric or duodenal ulcers, hepatic disease, or severe lactose intolerance).	Patients with any conditions affecting absorption, distribution, or metabolism of the study medication	P
		inflammatory bowel disease	Y
		gastric ulcers	Y
		duodenal ulcers	Y
		hepatic disease	Y
		severe lactose intolerance	Y
	Patients with evidence of clinically significant, active gastrointestinal, renal, hepatic, respiratory, endocrine, or cardiovascular system disease (including history of life-threatening arrhythmias).	clinically significant gastrointestinal disease	P
		clinically significant renal disease	P
		clinically significant hepatic disease	P
		clinically significant respiratory disease	P

		clinically significant endocrine disease	P
		clinically significant cardiovascular disease	P
	Patients with a history of cancer (does not include basal or squamous cell carcinoma of the skin) treated within 5 years prior to study entry, or current evidence of malignant neoplasm, recurrent, metastatic disease. Males with localized prostate cancer requiring no treatment would not be excluded.	Patients with a history of cancer (does not include basal or squamous cell carcinoma of the skin) treated within 5 years prior to study entry	Y
		current evidence of malignant neoplasm, recurrent, metastatic disease	Y
		Males with localized prostate cancer requiring no treatment would not be excluded	N
	Known plan for elective surgery during the treatment period that would require general anesthesia and administration of neuromuscular blocking agents.		N
	Donation of blood or blood products during 30 days prior to Screening or plans to donate blood while participating in the study or within 30 days after completion of the study.		N
	Patients who are unwilling or unable to fulfill the requirements of the study.		N
	Known hypersensitivity to acetylcholinesterase inhibitors or memantine.		N
	Use of any prohibited prior or concomitant medications) Any condition that would make the patient, in the opinion of the investigator, unsuitable for the study.		P
	Involvement in any other investigational drug clinical trial during the preceding 3 months, or likely involvement in any other such trial during the course of this study.		N
	Patients taking concomitant antidepressant medication known to have significant anticholinergic effects, such as tricyclic antidepressants prescribed at doses recommended for the treatment of major depression.		Y
	Patients who cannot swallow or who have difficulty swallowing whole tablets.		Y
	Patients with fecal and/or urinary incontinence who are unable to cooperate with routine specimen collection.	fecal incontinence	Y
		urinary incontinence	Y

Supplementary Table 3. Results of trial simulation with random dropout

	Original trial		Control arm simulation	Two arm simulation			
			Proportional sampling	Proportional 1:1 matching		Proportional 1:3 matching	
**Arm**	**23 mg**	**10mg**	**10mg**	**23mg**	**10mg**	**23mg**	**10mg**
Number of subjects	963	471	196	17	20	17	60
Mean age	73.9	73.8	78.9±0.1	79.2±0.1	79.5±0.2	79.1±0.1	79.4±0.2
Gender							
Female	63.0%	62.4%	66.1%±0.1%	70.2%±0.6%	69.8%±0.7%	70.4%±0.6%	69.4%±0.6%
Male	37.0%	37.6%	33.9%±0.1%	29.8%±0.6%	30.2%±0.7%	29.6%±0.6%	30.6%±0.6%
Race							
White	73.5%	73.5%	73.3%±0.1%	70.8%±0.1%	70.3%±0.1%	70.8%±0.1%	70.2%±0.1%
Hispanic	7.0%	5.5%	2.1%±0.1%	8.3%±0.1%	7.9%±0.3%	8.3%±0.1%	8.1%±0.3%
Black	2.3%	1.9%	5.4%±0.1%	4.2%±0.1%	4.2%±0.3%	4.2%±0.1%	4.2%±0.2%
Asian & Other	17.2%	0.6%	19.2%±0.1%	16.7%±0.1%	17.6%±0.3%	16.7%±0.1%	17.6%±0.3%
Charlson Comorbidity Index	n/a		1.91±0.1	1.26±0.03	1.40±0.02	1.22±0.03	1.21±0.01
SAE[a] rates (patient with >= 1 SAE)	9.6%	8.3%	8.8%±0.1%	9.8%±0.6%	7.9%±0.2%	9.1%±0.6%	5.6%±0.1%
Mean SAE (average SAE per patient)	0.15	0.14	0.19±0.01	0.99±0.04	0.22±0.01	0.99±0.01	0.19±0.01

Supplementary Table 4. Results of trial simulation with dropout based on occurrence of SAE

	Original trial		Control arm simulation
			Proportional sampling
**Arm**	**23 mg**	**10mg**	**10mg**
Number of subjects	963	471	196
Mean age	73.9	73.8	78.4±0.1
Gender			
Female	63.0%	62.4%	67.3%±0.2%
Male	37.0%	37.6%	32.7%±0.2%
Race			
White	73.5%	73.5%	73.5%±0.1%
Hispanic	7.0%	5.5%	1.9%±0.1%
Black	2.3%	1.9%	5.4%±0.1%
Asian & Other	17.2%	0.6%	19.2%±0.1%
Charlson Comorbidity Index	n/a		1.37±0.1
SAE[a] rates (patient with >= 1 SAE)	9.6%	8.3%	7.3%±0.1%
Mean SAE (average SAE per patient)	0.15	0.14	0.23±0.01
